# Psychotic disorder and educational achievement: a family-based analysis

**DOI:** 10.1007/s00127-015-1082-6

**Published:** 2015-06-23

**Authors:** Aleida Frissen, Ritsaert Lieverse, Machteld Marcelis, Marjan Drukker, Philippe Delespaul, GROUP Investigators

**Affiliations:** Department of Psychiatry and Psychology, Maastricht University Medical Centre, PO Box 616 (VIJV1), 6200 MD Maastricht, The Netherlands

**Keywords:** Psychosis, Cognition, Education, Trauma, Urbanicity

## Abstract

**Background:**

Early social and cognitive alterations in psychotic disorder, associated with familial liability and environmental exposures, may contribute to lower than expected educational achievement. The aims of the present study were to investigate (1) how differences in educational level between parents and their children vary across patients, their healthy siblings, and healthy controls (effect familial liability), and across two environmental risk factors for psychotic disorder: childhood trauma and childhood urban exposure (effect environment) and (2) to what degree the association between familial liability and educational differential was moderated by the environmental exposures.

**Methods:**

Patients with a diagnosis of non-affective psychotic disorder (*n* = 629), 552 non-psychotic siblings and 326 healthy controls from the Netherlands and Belgium were studied. Participants reported their highest level of education and that of their parents. Childhood trauma was assessed with the Dutch version of the Childhood Trauma Questionnaire-Short Form. Urban exposure, expressed as population density, was rated across five levels.

**Results:**

Overall, participants had a higher level of education than their parents. This difference was significantly reduced in the patient group, and the healthy siblings displayed intergenerational differences that were in between those of controls and patients. Higher levels of childhood urban exposure were also associated with a smaller intergenerational educational differential. There was no evidence for differential sensitivity to childhood trauma and childhood urbanicity across the three groups.

**Conclusion:**

Intergenerational difference in educational achievements is decreased in patients with psychotic disorder and to a lesser extent in siblings of patients with psychotic disorder, and across higher levels of childhood urban exposure. More research is required to better understand the dynamics between early social and cognitive alterations in those at risk in relation to progress through the educational system and to understand the interaction between urban environment and educational outcomes.

## Introduction

Cognitive alterations are core features during all phases of psychotic disorder, including premorbid state, onset, and longitudinal course [1–5]. Cognitive alterations are associated with disabilities in everyday functioning [6]. The origin of cognitive alterations remains unclear, and may be confounded by motivational factors [7]. Nevertheless, as cognitive alterations are present in the premorbid phases of psychosis [4, 5] and unaffected siblings show impaired cognitive functioning [8–10], developmental and genetic origins are likely.

Other substantiations for a developmental route come from a large Swedish birth cohort which showed lower level of school performance was associated with the development of schizophrenia [11]. Early cognitive alterations may thus contribute to alterations in educational achievement. However, other studies on scholastic achievement did not find such a clear association with the development of psychotic disorder. Not being in the expected class at age 14 predicted future hospital-treated disorders, but not specifically psychotic disorder [12] and poor school performance in art and handicrafts, but not academic performance was a risk factor for schizophrenia [13].

Investigating cognitive alterations in relation to educational achievement is important as it may shed light on the early origins of social disability associated with cognitive alterations in psychotic disorder. A range of non-cognitive factors are known to influence school performance and educational outcomes, socioeconomic status [14], and obstetric/perinatal complications [15–17] being well-known examples.

Few studies [1, 11] on cognitive or scholastic performance in psychotic disorder included data on educational performance of the parents. Parental educational level is required in order to interpret actual educational level in relation to the expected educational level as predicted by parental education [1]. No studies on educational performance included the unaffected siblings of patients with psychotic disorder, which similarly will increase the accuracy of interpreting actual versus expected educational level. As far as we are aware, it remains unknown whether unaffected siblings of patients with a psychotic disorder perform below the expected level in school. Furthermore, cognitive functioning in psychotic disorder is associated with sociodemographic factors, such as childhood trauma and stress [3] and urban habitat [18]. Stress and altered functioning of the hypothalamic–pituitary–adrenal (HPA) axis, with harmful effects of stress and glucocorticoids on the brain is a possible explanation for this association [19]. As these factors may thus impact the association between cognitive alterations and educational achievement, inclusion of these in the analyses may be profitable.

The aim of the present study was to investigate (1) how differences in educational level between parents and their children vary across patients, their healthy siblings and healthy controls (effect familial liability), and across two environmental risk factors for psychotic disorder: childhood trauma and childhood urban exposure (effect environment) and (2) to what degree the association between familial liability and educational differential was moderated by the environmental exposures (childhood trauma and childhood urbanicity). It was hypothesized that patients had lower levels of education compared to their parents, than controls in comparison with their parents, and that the healthy siblings were in between the controls and the patients in this respect. Childhood urban exposure and childhood trauma were hypothesized to negatively impact the educational differential. Thus, it was expected that in the patient group, and to a lesser degree in the sibling group, both environmental exposures would negatively influence the educational difference.

## Materials and methods

### Participants

Data pertain to baseline measures of an ongoing multisite, longitudinal, naturalistic cohort study, the Dutch national Genetic Risk and Outcome in Psychosis (GROUP) project [20]. Because data on parental education were not collected for the region Groningen, this region was excluded from the analysis. Participants younger than 23 years of age were also excluded from the analysis, because it is less likely that they would have completed their educational potential [21]. The sample thus consisted of 629 patients diagnosed with a non-affective psychotic disorder, 552 of their siblings, and 326 unrelated healthy control subjects from the general population from the Netherlands and Belgium. Controls were selected through a system of random mailings to addresses in the catchment areas of the cases. Exclusion criteria for controls were lifetime psychotic disorder and having a first degree relative with a lifetime psychotic disorder. The full selection procedure and in- and exclusion criteria have been described previously [20].

The study was approved centrally by the Ethical Review Board of the University Medical Centre Utrecht. Written informed consent was obtained from all subjects after they (1) read a document with detailed information about the nature and possible consequences of the study; (2) had verbally discussed any possible concerns with the researcher; and (3) had provided clear indication that they had understood the procedure. In the Netherlands, adult patients with mental illness are considered participating citizens who have the right to make independent informed decisions including the autonomous decision to participate in research; therefore, consent of relatives was not sought.

### Education

In the Netherlands, most schools are state schools which are organized in primary, secondary, and tertiary education tiers. From 4 until 12 years of age, all children receive primary education. After 12 years, children attend one of four levels of secondary education (low, intermediate, high preparatory vocational, and pre-university); each level requires increasing intellectual and scholastic abilities. After passing the exams in secondary education, there are three possible levels of tertiary education (intermediate professional education, higher professional education, and university).

Participants were asked to report their highest level of education and that of their parents, also if not completed. This conservative strategy was chosen as subjects with psychotic disorder are less likely to complete their education because of the emergence of psychotic symptoms [22] (mean age of onset was 25.0 in this study sample with a standard deviation of 6.5). Education was classified across 7 categories: Level 1: no education; Level 2: primary school; Level 3: lower secondary education; Level 4: intermediate professional education; Level 5: higher secondary education; Level 6: bachelor degree or equivalent (higher professional education); and Level 7: university (master degree).

### Childhood trauma

Childhood trauma was assessed with the Dutch version of the Childhood Trauma Questionnaire Short Form (CTQ) [23]. The short CTQ consists of 25 items rated on a 5-point Likert scale (1 = never true to 5 = very often true) enquiring about traumatic experiences in childhood. Five types of childhood maltreatment were assessed: emotional, physical and sexual abuse, and emotional and physical neglect, with five questions covering each type of trauma. The mean score for all 25 items was divided in tertiles (low, medium, and high childhood trauma). The latter was used as the primary variable reflecting childhood trauma in the analyses. CTQ data were missing for 353 persons (23 % missing data).

### Level of childhood urbanicity

A historical population density record was generated for each municipality from 1930 onwards using historical data from Statistics Netherlands and the equivalent database in Belgium [24, 25]. When data was not available, missing data were calculated by linear extrapolation between two subsequent time-points. When historical names of municipalities disappeared from historical records (e.g., due to city mergers) the available data from the agglomerate city were used. Subjects were asked to describe where they had lived at birth, between ages 0 and 4; between 5 and 9; 10–14; 15–19; 20–39; 40–59 years; and 60+ up to the actual age. This resulted in a number of records for each subject, containing locations by age period. For each of these records, we computed the average population density (by square kilometer, excluding water) of the municipality for the matching periods. Average population density over the period was categorized in accordance with the Dutch CBS urbanicity rating (1 ≤ 500/km^2^; 2 = 500–000/km^2^; 3 = 1000–1500/km^2^; 4 = 1500–2500/km^2^; 5 = 2500+/km^2^). The periods 0–4, 5–9, and 10–14 years were collapsed to produce average urbanicity exposure between 0 and 14 years, rounded to the nearest whole number. Urbanicity data was missing for 132 persons (9 % missing data).

### Statistical analyses

All analyses were performed using Stata 12 [26]. The main outcome variable was difference in educational level between the subject and highest level of education among the two parents (difference in education level (edu-dif) was calculated as the highest parental education—education subject).

In order to test whether edu-dif varied across patients with psychotic disorder, their healthy siblings and healthy controls and also across categories of childhood trauma and childhood urbanicity, multilevel linear regression models were fitted with edu-dif as the dependent variable and group status (patient, sibling, or control), childhood trauma (low, medium, and high childhood trauma), and childhood urbanicity (5 levels of childhood urbanicity) as independent variables. Multilevel regression analysis was used to account for clustering of repeated measures within families, using the Stata XTMIXED command, with family ID modeled as the macro level. The regression coefficient (*B*) represents the effect size of the predictors and can be interpreted as the estimates in equivalent unilevel linear regression analysis. All independent variables were entered together as categorical variables recoded into dummy variables (model 1). In a separate analysis, independent variables were entered together as continuous variables in order to obtain linear associations (model 2). Age, sex, and ethnicity were added as possible confounders. Age was added as confounder given that education, as well as access to education, may change over time. For the same reason, sex and ethnicity were added as (possible) confounders.

In order to study to what degree associations between edu-dif on the one hand and childhood trauma and childhood urbanicity on the other varied between patients, siblings, and controls, two-way interactions between childhood trauma and group and between childhood urbanicity and group were added to the model of edu-dif separately (childhood trauma and childhood urbanicity are both variables with n categories, entered as a continuous variable and as n-1 dummy variables). In the case of significant interaction, stratified effect sizes were calculated by linear combination of the effects from the model containing the main effect and the interaction term using the Stata MARGINS routine.

## Results

### Participants

The total sample consisted of 629 patients with a diagnosis of non-affective psychotic disorder, 552 of their siblings and 326 control subjects. Sociodemographic and clinical characteristics of the final sample are summarized in Table 1. The patients were younger and more often were male. The percentage of non-whites was higher in the patient group and the overall CTQ score and childhood urbanicity score were higher in the patient group. Patients and siblings had a significantly lower level of education than controls (controls: mean 4.42, SD 1.31; siblings: mean 4.24, SD 1.43; patients: mean 3.76, SD 1.48; controls compared to patients: *p* < 0.001; controls compared to siblings: *p* = 0.027). Parental education was significantly higher in the siblings compared to controls (controls: mean 3.28, SD 1.52; siblings: mean 3.81, SD 1.71, controls compared to siblings *p* = 0.004), and similarly directionally higher in the patients (patients: mean 3.58, SD 1.77, controls compared to patients *p* = 0.064).

**Table 1:** Subject demographics

	Patients (*n* = 629)	Siblings (*n* = 552)	Controls (*n* = 326)
Age (±SD)	30.8 ± 7.0	31.6 ± 7.4	35.6 ± 9.0
Sex *n* (%), male	484 (77.0)	248 (44.9)	131 (40.2)
Ethnicity			
Caucasian *n* (%)	484 (77.0)	462 (83.7)	299 (91.7)
Other *n* (%)	145 (23.1)	90 (16.3)	27 (8.3)
Childhood urbanicity^a^	2.9 ± 1.6	2.7 ± 1.5	2.7 ± 1.6
CTQ (±SD)	1.6 ± 0.5	1.4 ± 0.4	1.4 ± 0.4
Education *n* (%)			
No education and primary school	7 (1.2)	4 (0.8)	2 (0.6)
Lower secondary education	154 (25.0)	69 (13.1)	26 (8.1)
Intermediate professional education	143 (23.3)	129 (24.5)	68 (21.1)
Higher secondary education	80 (13.1)	38 (7.2)	34 (10.6)
Bachelor or equivalent	132 (21.5)	165 (31.4)	122 (37.9)
Master or equivalent	99 (16.1)	121 (23.0)	70 (21.7)
Highest parental education *n* (%)			
No education and primary school	57 (9.8)	28 (5.5)	17 (5.3)
Lower secondary education	159 (27.2)	146 (28.2)	124 (39.0)
Intermediate professional education	97 (16.6)	75 (14.5)	53 (16.7)
Higher secondary education	34 (5.8)	25 (4.8)	28 (8.8)
Bachelor or equivalent	121 (20.7)	132 (25.5)	67 (21.1)
Master or equivalent	116 (19.9)	112 (21.6)	29 (9.1)
Diagnosis *n* (%)			
Schizophrenia/schizophreniform	456 (73.2)		
Schizoaffective disorder	74 (11.9)		
Brief psychotic disorder	10 (1.6)		
Delusional disorder	8 (1.3)		
Substance-induced psychotic disorder	5 (0.8)		
Psychotic disorder NOS	55(8.8)		
Psychotic disorder due to medical condition	1 (0.2)		
Mood disorder	11 (1.8)		
Delirium	1 (0.2)		
Substance-related disorder	1 (0.2)		

*CTQ* childhood trauma questionnaire

^a^Five levels of urbanicity/population density *1* <500 inhabitants/km^2^;* 2* 500–1000 inhabitants/km^2^;* 3* inhabitants 1000–1500/km^2^;* 4* inhabitants 1500–2500/km^2^;* 5* 2500+/km

### Educational difference and main effects of group, childhood trauma, and childhood urbanicity

**Fig. 1:**
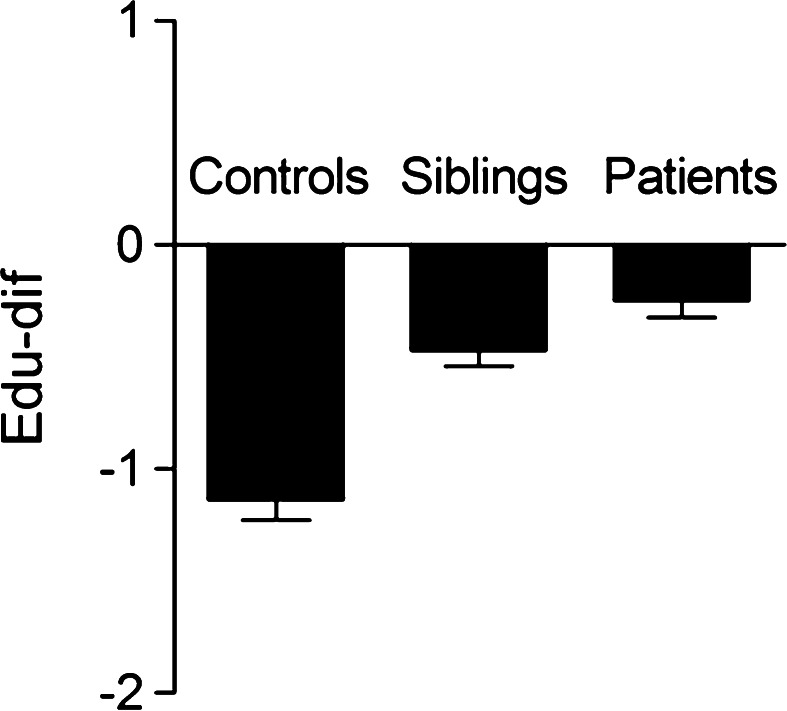
Educational difference (edu-dif) between highest level of parental education and subject education in controls, siblings, and patients. Represented values are population means and standard error of the mean

In all three groups, edu-dif had a negative value (Table 2; Fig. 1), indicating that participants had a higher level of education than their parents. There was a significant association between group (linear trend) and edu-dif (*B* = 0.36, *p* < 0.001). Edu-dif was significantly less negative in the patient group than in the control group (*B* = 0.77, *p* < 0.001), while siblings were in between the controls and the patients (*B* = 0.49, *p* = 0.001). The effect of maternal and paternal education was similar (maternal education: *B* linear trend = 0.36, *p* < 0.001, patients: *B* = 0.77, *p* < 0.001, siblings: *B* = 0.43, *p* = 0.003; paternal education: *B* linear trend = 0.34, *p* < 0.001, patients: *B* = 0.77, *p* < 0.001, siblings: *B* = 0.54, *p* < 0.001).

**Table 2:** Educational difference between highest level of parental education and subject education

	*n*	Mean (SD)	*B* (*p*)^a^	CI	*B* linear trend (*p*)^b^	CI
Group						
Controls	315	−1.14 (1.57)				
Siblings	510	−0.47 (1.61)	0.49 (0.001)	0.21 to 0.76		
Patients	581	−0.25 (1.75)	0.77 (<0.001)	0.48 to 1.06	0.36 (<0.001)	0.21 to 0.50
Childhood trauma						
Low	497	−0.73 (1.56)				
Medium	364	−0.61 (1.61)	0.07 (0.56)	−0.16 to 0.30		
High	416	−0.46 (1.67)	0.12 (0.34)	−0.12 to 0.36	0.07 (0.279)	−0.05 to 0.19
Urbanicity^c^						
Level 1	462	−0.82 (1.67)				
Level 2	321	−0.47 (1.61)	0.43 (0.002)	0.16 to 0.71		
Level 3	159	−0.17 (1.66)	0.68 (<0.001)	0.33 to 1.03		
Level 4	173	−0.47 (1.61)	0.49 (0.007)	0.13 to 0.84		
Level 5	335	−0.58 (1.72)	0.46 (0.002)	0.17 to 0.74	0.11 (0.002)	0.04 to 0.18

*B* represents the regression coefficients from multilevel linear regression analyses, adjusted for age, sex, and ethnicity

*SD* standard deviation, *CI* 95 % confidence interval

^a^Model 1: all independent variables entered as categorical variables recoded into dummy variables

^b^Model 2: all independent variables entered as categorical variables comprised in a linear model

^c^Five levels of urbanicity/population density* 1* <500 inhabitants/km^2^;* 2* 500–1000 inhabitants/km^2^;* 3* inhabitants 1000–1500/km^2^;* 4* inhabitants 1500–2500/km^2^;* 5* 2500+/km

**Fig. 2:**
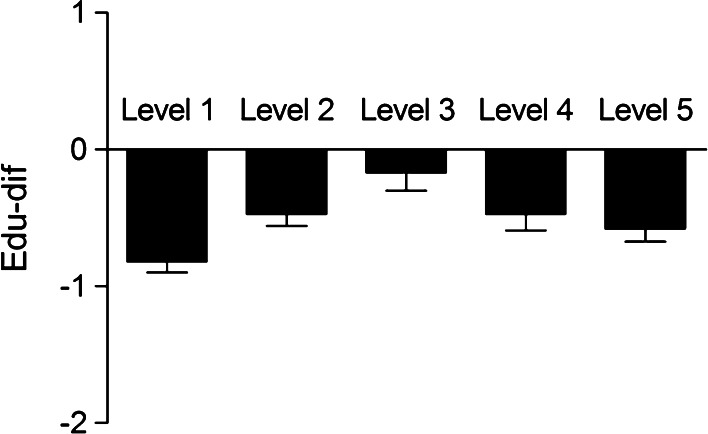
Educational difference (edu-dif) between highest level of parental education and subject education across five levels of urbanicity. Represented values are population means and standard error of the mean

Edu-dif was not associated with childhood trauma (B linear trend = 0.07, *p* = 0.279; Table 2). Childhood urbanicity was significantly associated with edu-dif (*B* linear trend = 0.11, *p* = 0.002), the main contrast being between urbanicity level 1 and the other 4 categories (Table 2; Fig. 2).

### Interaction between group and childhood trauma or childhood urbanicity

There was no evidence that associations between edu-dif and environmental risks differed by group (Table 3).

**Table 3:** Chi-square tests of interaction between group status (patients, sibling, or control) and childhood trauma or childhood urbanicity in the model predicting educational difference

	*χ* ^2a^	*Df* ^b^	*p*
Childhood trauma	2.37	2	0.31
Childhood trauma—dummy^c^	3.25	4	0.52
Urbanicity	1.67	2	0.43
Urbanicity—dummy^d^	7.50	8	0.48

Childhood trauma and childhood urbanicity are both categorical variables with n categories, entered both as a continuous variable and as *n* − 1 dummy variables

^a^Chi square

^b^Degrees of freedom

^c^Childhood trauma scores divided in tertiles (low, medium, high)

^d^Five levels of urbanicity/population density* 1* <500 inhabitants/km^2^;* 2* 500–1000 inhabitants/km^2^;* 3* inhabitants 1000–1500/km^2^;* 4* inhabitants 1500–2500/km^2^;* 5* 2500+/km

## Discussion

Educational difference (edu-dif) between subjects and their parents was studied in groups of psychotic patients, healthy siblings, and healthy controls. Overall, participants had a higher level of education than their parents. This difference was significantly less in the patient group and the healthy siblings were in between the controls and the patients. Further it was tested whether edu-dif was influenced by childhood trauma and childhood urbanicity, and if this was different for patients, siblings, and controls. Only childhood urban exposure had a significant influence on edu-dif: higher levels of childhood urban exposure were associated with a smaller increase in educational level. There was no evidence that this association differed for patients, siblings, or controls.

In all three groups, participants had a higher level of education than their parents. A reasonable explanation for this is development of education and growing availability of education over time. Patients and siblings had a smaller increase in educational level than the healthy controls. This is in line with previous research indicating early cognitive alterations in psychotic disorder [4, 5] and also with research on alterations in cognitive functioning in first-degree relatives of patients with psychotic disorder [8–10, 27]. Previous research on scholastic achievements in psychotic disorder is not consistent [28]. To the best of our knowledge, this is the first study to include siblings, thus shedding light on possible mediation by familial factors including genetic factors.

Our findings could be explained by several hypothesis. One plausible explanation is that early alterations in cognition, in patients and in siblings, may lead to problems in school functioning and eventually to lower levels of education. A second explanation is that altered social cognition, a specific part of cognitive functioning, that is not only impaired in psychotic disorder [29] but also in persons at genetic and clinical high risk for psychosis [30–32], elevates stress and lowers school performance. A third explanation is that the siblings of patients with a psychotic disorder receive less parental attention and guidance through their school development, as a negative consequence of the worries and attention focussed on the (pre)psychotic sib.

We did not find childhood trauma to influence intergenerational educational differential, in contrast with existing literature. Childhood trauma is associated with lower academic performance [33, 34], adverse effects on educational attainment [35], and academic delay [34]. Boden and colleagues [14] also found that physical and sexual abuse negatively affected educational achievements, but this was largely explained by social, family, and individual factors, after taking parental and maternal education into account. In this study, educational level was assessed relative to parental education, which could explain why there was no significant difference.

To our knowledge, there are no other studies on childhood urban exposure and scholastic achievements. However, factors associated with the urban environment and school performance have been examined previously and are in line with our results: urban noise exposure has a negative effect on school performance [36] and lower perceived safety of schools and neighborhoods deteriorates school performance [37]. Another explanation is the disappearance of small village schools and the subsequent emergence of larger schools with more pupils [38]. For pupils, such a larger scaled social study environment may have negative effects on school performance.

Childhood urbanicity and childhood trauma were not differentially associated with educational differences across psychotic patients, siblings, and in controls. This makes it unlikely that childhood trauma and childhood urbanicity influence the effect of group on educational difference, in a positive or negative way.

### Methodological considerations

Some limitations need to be addressed. The cross-sectional nature of the current data makes it impossible to establish a causal relationship between group status, childhood trauma, or childhood urbanicity and edu-dif; this was not the purpose of the study. Second, highest level of started education was studied, regardless of whether or not this was completed. This conservative approach was chosen because patients with psychotic disorder are less likely to complete education after the onset of psychotic symptoms [22]. Thus, studying completed education is more likely to measure the effect of psychotic symptoms, and not of early cognitive alterations, on educational achievement. Another approach could have been to compare grades in for example, primary education; however, grading systems may have changed over time and not all schools may use the same grading system, making this an inaccurate measure. Further, not all factors that could possibly affect school performance were taken into account, for example, family income and other non-educational factors associated with socioeconomic status, or obstetric and perinatal complications. However, school attendance in the Netherlands is not dependent on income as there is universal-free schooling. Fourth, the higher level of parental education for the siblings and at trend-level for the patients may have been the result of underlying selections, which could have influenced our results. However, the regression coefficients for the difference in parental education are smaller than the regression coefficients of edu-dif, which makes it unlikely that possible selection is the only explanation of the observed difference in edu-dif. Finally, the participants grew up in the Netherlands and Belgium which can be described as relatively safe and developed countries; in other counties, urban–rural discrepancies may be more prominent.

## Conclusion

Patients with psychotic disorder, and to a lesser extent their healthy siblings, had a smaller increase in educational level compared with their parents than healthy controls. Although these group differences cannot be used to identify groups at high risk, it does provide a general perspective in thinking about intergenerational processes in educational achievement in the context of risk for psychosis. More work is required to better understand the dynamics between early social and cognitive alterations in those at risk in relation to progress through the educational system. Higher levels of childhood urbanicity were also associated with a smaller increase in educational level. As more people are residing in urbanized areas [39], more work is required to understand the interaction between urban environment and educational outcomes including school size, class size, level of individual educational support, and class dynamics.

## Appendix

Genetic Risk and Outcome of Psychosis (GROUP) Investigators: Richard Bruggeman^a^, Wiepke Cahn^b^, Lieuwe de Haan^c^, René Kahn^b^, Carin Meijer^c^, Inez Myin-Germeys^d^, Jim van Os^d,e^, Durk Wiersma^a^

^a^University Medical Center Groningen, Department of Psychiatry, University of Groningen, The Netherlands, ^b^University Medical Center Utrecht, Department of Psychiatry, Rudolf Magnus Institute of Neuroscience, The Netherlands, ^c^Academic Medical Centre University of Amsterdam, Department of Psychiatry, Amsterdam, The Netherlands, ^d^Maastricht University Medical Centre, South Limburg Mental Health Research and Teaching Network, EURON, Maastricht, The Netherlands, ^e^King’s College London, King’s Health Partners, Department of Psychosis Studies, Institute of Psychiatry, London, United Kingdom.
